# Power Performance Verification of a Wind Farm Using the Friedman’s Test

**DOI:** 10.3390/s16060816

**Published:** 2016-06-03

**Authors:** Wilmar Hernandez, José Luis López-Presa, Jorge L. Maldonado-Correa

**Affiliations:** 1 Departamento de Ciencias de la Computación y electrónica, Universidad Técnica Particular de Loja, Campus de la Universidad Técnica Particular de Loja, Calle San Cayetano Alto s/n, Loja 1101608, Ecuador; jlmaldonado7@utpl.edu.ec; 2 Departamento de Ingeniería Telemática y Electrónica, Universidad Politécnica de Madrid, Madrid 28031, Espana; joseluis.lopezp@upm.es

**Keywords:** SCADA system, wind farm power performance, nonparametric statistical tests

## Abstract

In this paper, a method of verification of the power performance of a wind farm is presented. This method is based on the Friedman’s test, which is a nonparametric statistical inference technique, and it uses the information that is collected by the SCADA system from the sensors embedded in the wind turbines in order to carry out the power performance verification of a wind farm. Here, the guaranteed power curve of the wind turbines is used as one more wind turbine of the wind farm under assessment, and a multiple comparison method is used to investigate differences between pairs of wind turbines with respect to their power performance. The proposed method says whether the power performance of the specific wind farm under assessment differs significantly from what would be expected, and it also allows wind farm owners to know whether their wind farm has either a perfect power performance or an acceptable power performance. Finally, the power performance verification of an actual wind farm is carried out. The results of the application of the proposed method showed that the power performance of the specific wind farm under assessment was acceptable.

## 1. Introduction

After a wind farm is built, the power performance of each wind turbine must be verified in accordance with the international standard IEC61400-12-1 [1]. However, when restrictions of an economic or technical nature appear, then it is recommended to use the data from the Supervisory Control and Data Acquisition (SCADA) system to verify the power curve of the wind turbines [2,3]. Nevertheless, attention should be paid to the fact that wind speed measurements collected by the nacelle anemometer do not accurately represent the free-stream wind speeds experienced by the rotor. However, this problem can be addressed if the measurements can be adjusted [3].

In the scientific literature, several research works have been published in which data from the SCADA system are used to carry out the performance monitoring of wind turbines. In [4], research was carried out in order to create a model of the power output of each wind turbine during fault-free operation from the data of the SCADA system. In [5], the data from the SCADA system were used to obtain different models based on artificial intelligence techniques for the monitoring of the power output of a wind farm. In [6], historical data of the SCADA system were used for the construction of models for analyzing power curves of wind turbines. In [7], the SCADA data were effectively used in the process of tuning a wind farm and to provide early warnings of possible failures. In that paper, historical wind turbine data were used to construct reference curves of wind power, rotor speed, and blade pitch angle, with wind speed as an input variable. Furthermore, in [8] an effective method for processing raw SCADA data was presented. Also, in that paper the authors proposed an alternative condition monitoring technique based on investigating the correlations among relevant SCADA data.

Moreover, with respect to novel methods of estimation of the power output of wind farms, in [9] the authors presented a novel probabilistic technique that was based on the use of power probability distribution functions to estimate the power output of each wind turbine. In addition, in [9] the power output of the wind farm was estimated in a probabilistic manner, using the previously calculated distribution functions and assigning Poisson distribution as the statistical spatial distribution for wind speed over the wind farm. In [10], estimation using polynomial regression, polynomial regression by weighing, and splines have also been used. In [11], regression procedures have been applied by using logistic equations. Also, in order to carry out the modeling of the power curve of wind turbines, models based on data mining have been used in [12]. In that paper, fuzzy logic, neural networks, and models of order k closest neighbors were used.

Furthermore, in [13] Gaussian models and censored Gaussian models to approximate the power curve were proposed. In [14], the authors presented a multi-sensory system for fault diagnosis in wind turbines, combined with a data-mining solution for the classification of the operational state of the turbine. In [15], the authors explained the basic concepts of power curve, the different methodologies that are used for its estimation, and presented an approximation to the estimation of this curve by using a kernel method with multiple factors. Also, the use of the kernel method can be seen in [16]. In [17], the authors discussed the anomalies that appear on the power curve when an irregularity occurs in order to try to relate the probability of interruption of operation of wind turbines with the wind speed. In [18], heavy-tailed distributions have been used in order to analyze the problem of appearance of outliers when there are significant changes in wind speed. In [19], Copula theory is adopted to establish the probability distribution of correlated input random variables, and this theory is also applied for constructing the multivariate distribution function of wind speeds at different wind sites [20].

In addition, in [21], due to the fact that the data from the SCADA system records often contain significant measurement deviations, the authors presented a probabilistic method for eliminating outliers that was developed by using a copula-based joint probability model. In [22], SCADA data were used to demonstrate the applicability of a probabilistic model of a power curve for condition monitoring purposes that was developed by the authors. In that paper, the application of copulas to modeling the power curve of a wind turbine was developed. In [23], a comprehensive overview on the wind turbine power curve modeling techniques is presented. In that paper, several parametric and nonparametric modeling techniques that have been employed for wind turbine power curve modeling are presented in detail.

Needless to say, the abovementioned papers represent only a small quantity of the many published papers related to probability and SCADA usage.

In the present paper, a novel method of verification of the power performance of a wind farm is presented. The proposed method is based on a nonparametric statistical inference technique, the Friedman’s test [24,25]. This method uses the information from the sensors embedded in the wind turbines that is collected by the SCADA system to determine whether the power performance of the specific wind farm under assessment differs significantly from what would be expected. Here, the guaranteed power curve is used as one more wind turbine of the wind farm that is under assessment, and a multiple comparison method is used to investigate differences between pairs of wind turbines with respect to their power performance.

The power performance verification method presented in this paper was applied to an actual wind farm. Here, the wind farm under analysis was the Villonaco Wind Farm (VWF), which is located in the province of Loja in southern Ecuador, in the hilltop of the Villonaco [26,27]. The VWF is placed in a complex terrain. Wind farms placed in complex terrains have to operate under harsh and undesirable flow conditions that affect their performance. This type of terrain is affected by high variations of turbulence. In addition, in some cases, the value of the inflow angle can be undesirable due to steep slopes, and the geographic features have an extremely important influence on the wind shear. All of this, among other factors, justifies the need for novel procedures that allow wind farm owners to know whether their farms need maintenance or which turbines are performing below expectations.

In the scientific literature, there are several research papers that have been focused on wind farms placed in complex terrains. For example, in [28] a three-dimensional flow simulation is performed to investigate the wind flow in a wind farm in a mountainous area of complex terrain. The method presented in [28] can be applicable for optimal arrangements of turbines in the wind farm. In [29], the authors highlighted the importance of advanced computer-aided engineering tools in the cognitive communication process, involved in the wind resource assessment and wind farm design optimization. In [30], a novel optimization method is proposed to optimize the layout for wind farms in complex terrains. In [31], a model to improve the quality of wind and power production forecasts, especially in complex terrains, is presented.

The organization of this paper is as follows: Section 2 is devoted to general comments about the most important sensors of a wind turbine and the SCADA system. Section 3 makes general comments on the current wind farm power performance verification method. Section 4 is devoted to the problem formulation. Section 5 presents a procedure to determine which wind turbines are producing a different outcome from what is expected. Section 6 is devoted to carrying out the power performance verification on an actual wind farm using nonparametric statistical inference. Lastly, Section 7 is devoted to the conclusions of this paper.

## 2. Some General Comments about the Most Important Sensors of a Wind Turbine and the SCADA System

The previous section was aimed at making general comments on some research papers that present both well-known and new techniques for verifying the power curve of wind turbines. Overall, the abovementioned papers were focused mainly on the following topics: power curve modeling, power curve estimation, condition monitoring of wind turbines, correlation among relevant SCADA data, and outlier elimination, among others. Statistical inference techniques and artificial intelligent techniques have been used.

However, before going on to present the power performance verification method proposed in this paper, it is important to make some general comments about the most common sensors embedded in wind turbines and the operation of the SCADA system.

To this end, it is important to highlight that sensors play a fundamental role in wind turbines. They allow wind farm operators to increase the efficiency of their wind turbines and energy production and to ensure reliability. In order to achieve this aim, the SCADA system continually processes the information coming from sensors embedded in the wind turbine and on the nacelle. In accordance with [32], some of the measured parameters that are of interest are the following:

- Wind speed and direction
- Rotor and generator speed
- Temperature (ambient, bearings, gearbox, generator, nacelle)
- Pressure (gearbox oil, cooling system, pitch hydraulics)
- Pitch and yaw angle
- Electrical data (voltage, current, phase)
- Vibrations and nacelle oscillation

In addition, examples of sensors used in wind turbine control systems are the following [33]:

- anemometers
- wind vane
- rotor speed sensors
- electrical power sensors
- accelerometers
- load sensors
- pitch position sensors
- temperature sensors
- oil level indicators
- hydraulic pressure sensors, *etc.*

However, the information coming from the sensors is usually corrupted by noise and interference, and it is necessary to carry out a filtering process before using the data to make decisions on the performance of the wind turbine. Examples of robust filtering techniques that have been used to diminish unwanted information in the performance of the turbine can be found in [34].

On the other hand, the SCADA system, which very often is designed by the same wind turbine manufacturer, sends the information to the wind farm management office and controls the operation of the wind turbines.

Here, it is important to point out that the SCADA system is designed to reject unwanted information in an optimal manner. Nevertheless, the SCADA system not only collects satisfactory performance data, but also collects information about faults and malfunctioning of the wind turbine operation. This information does not represent noise and interference that corrupt the electrical signals of the sensors. Therefore, this information appears on the actual power curve of the wind turbines as anomalous data and outliers, and does not represent the true power curve of the wind turbine. This is the reason why it is necessary to carry out a further filtering process, in which the person responsible for verifying the power performance of the wind turbines and of the wind farm in general can analyze only the most representative data.

In this sense, this paper is aimed at presenting a method for verifying the power performance of a wind farm by taking into consideration the data from the SCADA system. Here, the anomalous data and outliers are eliminated by using a simple, easy to implement process; afterwards, the power performance of the wind farm is verified by using a novel method based on the Friedman’s test [24,25].

## 3. Some General Comments on the Current Method for the Power Performance Verification of a Wind Farm

According to [32], the International Electrotechnical Commission (IEC) has bundled together under the number 61,400 several standards for different sectors of the wind energy. For example, five of these standards are the following:

- IEC 61400-1 Design Requirements
- IEC 61400-2 Design Requirements of Small Wind Turbines
- IEC 61400-3 Design Requirements for Offshore Wind Turbines
- IEC 61400-11 Acoustic Noise Measurements Techniques
- IEC 61400-12-1 Power Performance Measurements of Electricity Producing Wind Turbines

Overall, IEC 61400-12-1 encompasses the procedure for power performance assessment of wind turbines, including measurement instrumentation and data analysis. IEC 61400-12-1:2005 specifies a procedure for measuring the power performance characteristics of a single wind turbine and applies to the testing of wind turbines of all types and sizes connected to the electrical power network. It also describes a procedure to be used to determine the power performance characteristics of small wind turbines when connected to either the electric power network or a battery bank. New versions such as IEC 61400-12-2:2013 specify a procedure for verifying the power performance characteristics of a single electricity-producing, horizontal axis wind turbine, which is not considered to be a small wind turbine per IEC 61400-2. This standard is intended to be used when the specific operational or contractual specifications may not comply with the requirements set forth in IEC 61400-12-1:2005 [1].

In accordance with [1], the parameters that are taken into consideration for the assessment of the power curve of the wind turbine are the following:

- Test site
- Test equipment
- Measurement procedure
- Derived results.

In short, the test site is the position of the wind turbine under assessment and its vicinity. Here, a meteorological mast must be installed near the wind turbine to measure wind speed and direction. This process involves an experimental site calibration, which requires the installation of one more meteorological mast, anemometers, wind direction sensors, and a data acquisition system along with a data logger. In addition, equipment is needed for carrying out the following measurements: electric power, wind speed, wind direction, air density, rotational speed and pitch angle, blade condition, and wind turbine control system.

Furthermore, regarding the measurement procedure, it is a well-known fact that companies that carry out the power performance verification of wind turbines keep the know-how of their measurement procedures as classified information. Therefore, it is not very common to find detailed step-by-step information about this procedure. However, taking into consideration the information given in the standard, technicians know the variables that must be measured, the sampling frequency for each type of measurement, the number of measurement points for each type of measurement, the type of statistical analysis to be applied to the collected data, and the type of data that must be rejected.

Moreover, with regard to the derived results, the standard also defines clearly how the data must be normalized, how the power curve must be determined, how the annual energy production must be determined, how the power coefficient must be determined, and, finally, how the reporting format has to be done.

To sum up, for a wind farm owner, the application of the methodology based on the IEC standard to carry out a power performance verification method of the wind turbine can be expensive. Furthermore, it is worth noting that in very large wind farms (on the order of some kilometers of extension) carrying out the power performance verification according to IEC standards could be impossible due to the huge cost. Therefore, due to the cost of the standard power performance verification method, the wind farm owner cannot do it very often. This is the reason why it is important to propose inexpensive alternative methods. As will be seen in the next sections, the method proposed in this paper only needs the data collected by the SCADA system.

## 4. Problem Formulation and Some Wind Farm Power Performance Statements

Consider a wind farm WF as a set {WT*_i_*} of *n* wind turbines WT*_i_* that are placed either onshore or offshore and interconnected through an electrical system, and whose main purpose is to produce electricity by converting kinetic energy from the wind into electrical power.

**Problem Formulation**.  *Given set WF of n WT_i_, verify the power performance of WF by using the information of the SCADA system to compare the power performance of each WT_i_ with the guaranteed power curve for i* = 1,…, *n*.

Next, a practical, convenient definition of the power performance of a WF is introduced. Then, this definition is used to mathematically formalize the power performance of a WF under test in terms of the median of the power output of the wind turbines (WTs) of such a WF and the guaranteed power curve (GPC) of the WTs.

**Definition 1**.  A wind farm WF is said to have a perfect power performance if, considering the GPC of the WTs as one more WT, there are not significant differences among the power curves of the WTs (including the GPC) at some α significance level. However, if the power performance of the WF is not perfect but there are not significant differences between the power curve of each individual WT and the GPC for at least 80% of the WTs at some α significance level, and also the other 20% of the WTs are in operation but they need some minor technical adjustments in order to increase their electricity production in some intervals of wind speed, then it is said that the WF has an acceptable power performance.

Let $P\in {ℛ}_{>0}^{m\times n}$ (${ℛ}_{>0}$: set of positive real numbers) be an *m*-by-*n* matrix whose element $p_{h,i}$ is the power output of the *i*-th WT, i=1,…,n of the WF at the *h*-th measurement point of the GPC, h=1,…,m. *m* is the number of wind speed values of the GPC (*i.e.*, the measurement points) and *n* is the number of WTs of the WF. Let WT*_i_* be a random variable from a population with a completely unspecified probability distribution ${ℱ}_{i}$ and let wt*_i_* = $\left(p_{1,i},\ldots ,p_{m,i}\right)$ be the observed realization of WT*_i_*. In addition, let the power output of the GPC be a random variable from a population with probability distribution ${ℱ}_{GPC}$ that represents the GPC evaluated at each of the *m* measurement points. Moreover, the WF under testing consists of *n* independent WTs and there are k=n+1 independent sets of observations, one from each of the populations ${ℱ}_{1},\ldots ,{ℱ}_{n}$ and one from the population ${ℱ}_{GPC}$, where the size of the *j*-th random sample is equal to $n_{j}$, with j=1,…,k.

The data consist of $\overset{k}{\underset{j=1}{\sum }}n_{j}=N$ observations. The location parameter $\theta_{hj}$ for h∈{1,…,m} and j∈{1,…,n} is the median of the population ${ℱ}_{j}$ at the *h*-th measurement point, for h∈{1,…,m} and j=k (recall that ${ℱ}_{GPC}={ℱ}_{k}$) is the value of the GPC $x_{h}$. Also, $\theta_{hj}$ is unknown for h∈{1,…,m} and j=k. Furthermore, $\theta_{hj}>0$ and $n_{j}=n_{l}=m$ for all j,l∈{1,…,k}.

**Theorem 1**.  *The power performance of a WF is perfect if the null hypothesis* $H_{0}:\;{ℱ}_{1}=\;\cdots =\;{ℱ}_{n}={ℱ}_{GPC}$ *cannot be rejected at any specific α significance level*.

Theorem 1 is based on the Friedman’s test [24,25] and the null hypothesis asserts that the distributions ${ℱ}_{1},\ldots ,{ℱ}_{n},\;{ℱ}_{GPC}$ are the same for each WT*_i_*, i=1,…,n. According to Friedman [25], this test involves first ranking the data in each row of a two-way table and then testing to see whether the different columns of the resultant table of ranks can be supposed to have all come from the same universe. This test is made by computing from the mean ranks for the several columns of a statistic, which tends to be distributed according to the chi-square distribution when the ranking is random, *i.e.*, when the factor tested has no influence. The Friedman’s test tests only for column effects after adjusting for possible row effects.

In [25], Friedman explained the test by presenting an example, and researchers that have used the test since then have followed the same steps as Friedman. The data and assumptions made in this section can be seen as the mathematical formalization of the power performance verification problem framed in the Friedman's test, and the procedure to compute the Friedman statistic is shown next in the proof of Theorem 1.

**Proof of Theorem 1**.  Taking into account the above assumptions, the null hypothesis for the *h*-th measurement point is that there are no differences among $\theta_{h1}\ldots \;\theta_{hk}$, h∈{1,…,m}, namely,

(1) $$H_{0}:\;\theta_{h1}=\;\cdots =\;\theta_{hn}=\theta_{hk}$$

and the alternative hypothesis is the following: $H_{1}:\;\exists \;u\neq v\;$with u,v∈{1,…,k} such that $\theta_{hu}\;\neq \theta_{hv}$. Also, in order to perform the test, the procedure is the following:

First, for each measurement point h∈{1,…,m} rank the k=n+1 observations from 1 to k.

Second, sum the ranks for each wind turbine WT*_i_*, i=1,…,n, and also for the GPC evaluated at the *m* measurement points.

Third, let the sums of the ranks for the *n* wind turbines and the GPC be $R_{1},\ldots ,R_{n}$ and $R_{k}$, respectively.

Fourth, the Friedman statistic [24,25] is given by

(2) $$S=\frac{12}{mk\left(k+1\right)}\left[\overset{k}{\underset{j=1}{\sum }}R_{j}^{2}\right]-3m\left(k+1\right)$$

Fifth, at the α significance level, reject $H_{0}$ (see Equation (1)) if $S\geq s_{\alpha }$, where $s_{\alpha }$ is the critical value, and, for m≥10 and k≥4, the critical region of size α is the upper portion of the chi-square distribution with k−1 degrees of freedom [24].

Sixth, and finally, if $H_{0}$ (see Equation (1)) cannot be rejected at the specified α significance level, then the power performance of the WF is perfect (see Definition 1).

Now, in accordance with Definition 1, if the power performance of the WF is not perfect, we must verify whether it is acceptable or not. In order to make that decision, a new proposition that follows Theorem 1 is presented as Corollary 1. This corollary is used for restating Theorem 1 for the special case that the WF is not perfect.

**Corollary 1**.  *If* $H_{0}$ *(see Equation (1)) is rejected at the α significance level but for at least 80% of the WTs the hypothesis*

(3) $$H_{0ik}:\;\theta_{hi}=\theta_{hk}\;\mathrm{for}\;i\in \left\{1,\ldots ,n\right\}\;\mathrm{and}\;h\in \left\{1,\ldots ,m\right\}$$

*cannot be rejected, then the power performance of the WF is acceptable (see Definition 1)*.

For the case where the WF is not perfect, a procedure to compare the power performance of the wind turbines to determine which wind turbines did produce either a superior outcome or an inferior outcome has been devised. This procedure is presented in the next section.

## 5. Procedure to Determine Which Wind Turbines are Producing a Different Outcome from What Is Expected

At the end of the previous section, it was mentioned that a procedure to determine which wind turbines prevent the wind farm performance under assessment from being perfect was devised. A procedure of this type is necessary because it is important to know whether the wind farm owner has to take actions to adjust the parameters of any specific wind turbine in order to improve its power performance.

Before continuing, it is worth mentioning that the procedure proposed in this section should be applied only after having done what is mentioned in the previous section.

### 5.1. Proposed Procedure

First, for each one of the *m* measurement points, rank the k observations from 1 to k. Call this ranks $r_{ij}$, $i=1,\;\ldots ,\;n_{j}$ and j=1, …, k, where $n_{j}$ is the number of measurement points of the j-th wind turbine and the guaranteed power curve.

Second, define the following:

(4) $$T_{\cdot j}=\sum_{i=1}^{n_{j}}r_{ij}\;\mathrm{for}\;j=1,\;\ldots ,\;k$$

(5) $$\bar{R_{\cdot j}}=\frac{T_{\cdot j}}{n_{j}}\;\mathrm{for}\;j=1,\;\ldots ,\;k$$

(6) $$T_{\cdot \cdot }=\overset{k}{\underset{j=1}{\sum }}T_{\cdot j}$$

(7) $$N=\overset{k}{\underset{j=1}{\sum }}n_{j}$$

(8) $$\bar{R_{\cdot \cdot }}=\frac{T_{\cdot \cdot }}{N}$$

(9) $${\hat{\sigma }}_{e}^{2}=\frac{\sum_{j=1}^{k}\sum_{i=1}^{n_{j}}r_{ij}^{2}-\sum_{j=1}^{k}\frac{T_{\cdot j}^{2}}{n_{j}}}{N-k}$$

where $\bar{R_{\cdot 1}},\;\ldots ,\;\bar{R_{\cdot k}}$, for k=n+1, are the mean values of the ranks of the n wind turbines and the guaranteed power curve, respectively; and ${\hat{\sigma }}_{e}^{2}$ is the mean square error of the ranks.

Third, build the following set of all pairwise comparisons:

(10) $$\begin{array}{ccccc} \bar{R_{\cdot 1}}-\bar{R_{\cdot 2}} & \bar{R_{\cdot 1}}-\bar{R_{\cdot 3}} & \cdots & \bar{R_{\cdot 1}}-\bar{R_{\cdot 11}} & \bar{R_{\cdot 1}}-\bar{R_{\cdot 12}}\\ \bar{R_{\cdot 2}}-\bar{R_{\cdot 3}} & \bar{R_{\cdot 2}}-\bar{R_{\cdot 4}} & \cdots & \bar{R_{\cdot 2}}-\bar{R_{\cdot 12}} & \\ \cdots & \cdots & \cdots & & \\ \bar{R_{\cdot 10}}-\bar{R_{\cdot 11}} & \bar{R_{\cdot 10}}-\bar{R_{\cdot 12}} & & & \\ \bar{R_{\cdot 11}}-\bar{R_{\cdot 12}} & & & & \end{array}$$

Fourth, for a 100(1−α)% confidence interval use the following formula [35] for the difference between two wind turbines:

(11) $$\left(\bar{R_{\cdot i}}-\bar{R_{\cdot j}}\right)\pm q_{\alpha ,k,N-k}\sqrt{\frac{{\hat{\sigma }}_{e}^{2}}{2}\left(\frac{1}{n_{i}}+\frac{1}{n_{j}}\right)}\;\mathrm{for}\;i=1,\;\ldots ,\;n_{j}\;\mathrm{and}\;j=1,\;\ldots ,\;k$$

where $q_{\alpha ,k,N-k}$ is the 100(1−α)th percentile of the Studentized range distribution with parameter k and N−k degrees of freedom, where N is given by Equation (7).

Here, it is important to say that Equation (11) is due to the Tukey-Kramer method for multiple comparisons [35], and the application of this method to the case under study is to reject the null hypothesis

(12) $$H_{0}:\bar{R_{\cdot i}}=\bar{R_{\cdot j}}\;\mathrm{for}\;i\neq j$$

If

(13) $$\left|\bar{R_{\cdot i}}-\bar{R_{\cdot j}}\right|>q_{\alpha ,k,N-k}\sqrt{\frac{{\hat{\sigma }}_{e}^{2}}{2}\left(\frac{1}{n_{i}}+\frac{1}{n_{j}}\right)}$$

In this case, the statistic is given by [35]:

(14) $$TK=\frac{\left|\bar{R_{\cdot i}}-\bar{R_{\cdot j}}\right|}{\sqrt{{\hat{\sigma }}_{e}^{2}\left(\frac{1}{n_{i}}+\frac{1}{n_{j}}\right)}}$$

Fifth, and finally, at the α significance level, reject $H_{0}$ (Equation (12)) if $TK\geq tk_{\propto }$, where the constant $tk_{\propto }$ is the critical value. In other words, at the α significance level, reject $H_{0}$ (Equation (12)) if *p*-value = $P(TK\geq tk|H_{0}\;\mathrm{is}\;\mathrm{true})<\alpha$, where tk is the observed value of TK (Equation (14)).

After the application of the previous steps, at the α significance level, the pairs that do not have a significantly different power performance can be found.

## 6. Power Performance Verification of an Actual Wind Farm Using Nonparametric Statistical Inference

**Figure 1:**
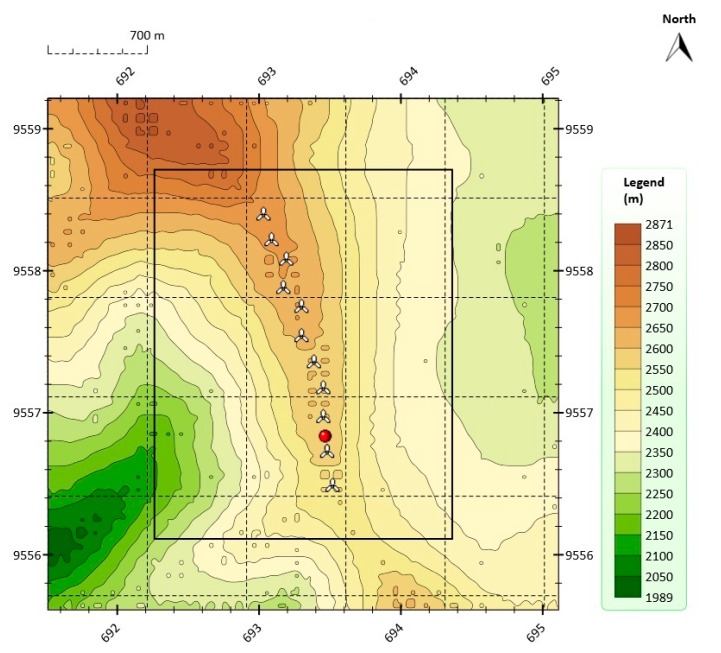
Orographic map of the VWF with wind turbine positions in UTM (Universal Transverse Mercator) coordinates.

As was already mentioned in Section 1, the wind farm under analysis was the Villonaco Wind Farm (VWF). The VWF consists of 11 × 1.5 MW Goldwind GW70, Permanent Magnet Direct Drive, IEC Class “S” wind turbine generators along a ridge approximately 6 km (aerial distance) to the west of the city of Loja. Figure 1 shows the orographic map of the VWF.

**Figure 2:**
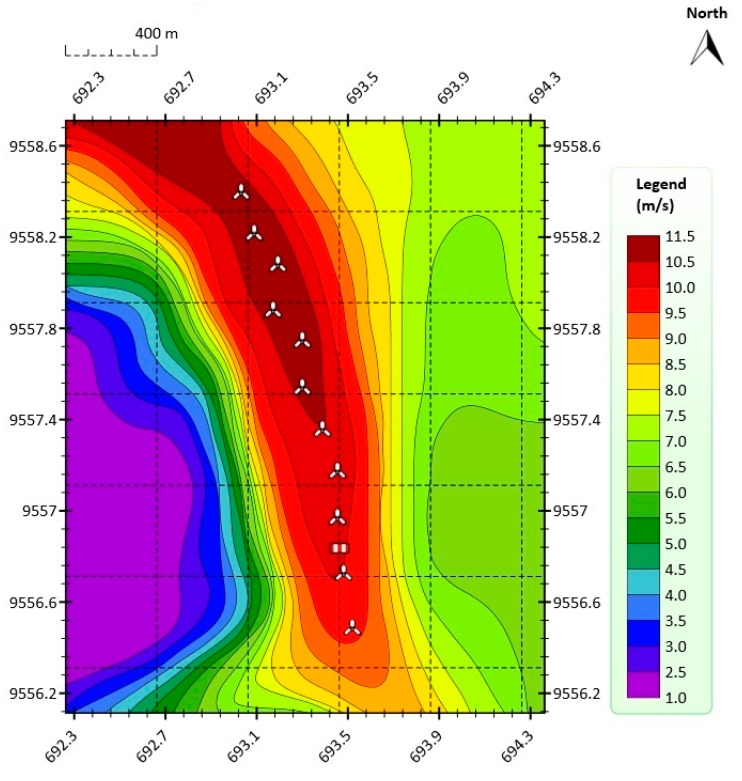
Annual average wind speed at 100 m AGL at the VWF in 2014.

Figure 2 shows the annual average wind speed at 100 m above ground level (AGL) at the VWF. The annual mean wind at 100 m AGL was greater than 10.5 m/s in the year 2014, which was the year in which the data were collected (from 1 January to 31 December).

In this paper, in order to obtain the orographic map shown in Figure 1 and the annual average wind speed map shown in Figure 2, the Meteodyn WT wind resource assessment software was used [36].

**Figure 3:**
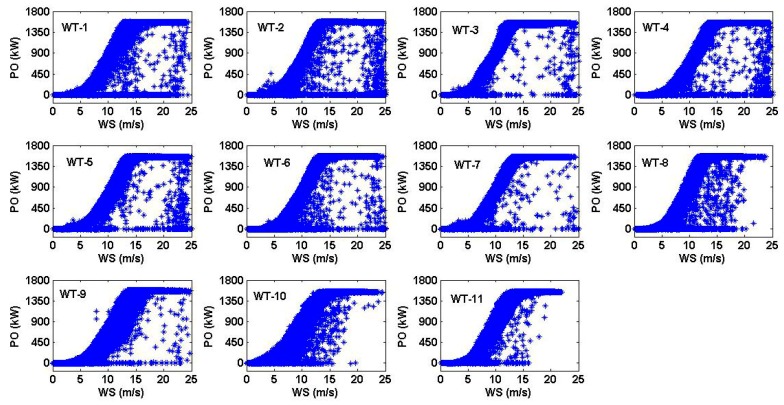
Scatter plot of the wind speed vs the power output of the 11 WTs of the VWF for 2014. WTi: *i*-th wind turbine, i∈{1,…,11}; PO: Power output; WS: Wind speed.

In order to verify the power performance of the VWF, in this paper the wind speed and power output information of each wind turbine were taken from the Goldwind SCADA system. The wind speed was measured by using the nacelle anemometer of each turbine, the hub height was equal to 65 m, and the sampling interval was equal to 10 min [1]. Figure 3 shows the scatter plot of the wind speed *versus* the power output of the 11 WTs of the VWF for the year 2014. The method that was used in this paper to find the most representative points of the power curves of the WTs is presented in Section 6.1.

### 6.1. Finding the Most Representative Points of the Power Output of Each Wind Turbine for Each Wind Speed

The scatter plot of the wind speed *versus* the power output of the wind turbine (WT) shows the relationship that exists between these two variables and proves that one variable is causing the other. However, when looking at all the scatter plots that are shown in Figure 3, it can be seen that there are outliers or extreme observations. These anomalous observation points can have many causes. For example, a WT may have suffered a transient malfunction, the SCADA system may have transmitted erroneous information at some specific time instant, some mistakes that are made by humans, faults in the performance of the WT during its operation, and so on.

On the other hand, sometimes outliers are not part of the so-called dirty data and they contain valuable information about the power performance of the WT and/or the data gathering and recording process carried out by the SCADA system. Therefore, the process of removing outliers should be carried out carefully.

In this sense, in this paper, in order to obtain the most representative points of the power output of the WTs (see Figure 3) and build the power curves that were compared with the guaranteed power curve (GPC), the following steps were followed:

First, the time intervals in which the registered values of power output were not in correspondence with the recorded wind speed were filtered out. Therefore, numerical values for which the power output was less than or equal to zero were discarded, because these values represent faults in the performance of the WT during its operation. Here, it is important to mention that at some wind speed values there were many samples, while at others there were no samples at all.

Second, for each wind speed value that was registered by the SCADA system, the three most significant power output values of each of the 11 WTs were found. In order to do this, the power output values were considered as points of the Euclidean 2D space and the most significant point for each specific wind speed value was the one whose Euclidean distance to the other points was the shortest.

**Definition 2**.  *(Three most significant points of the power output of a WT at a specific wind speed value, for the case in which all the power output values are different from each other). Let* $Z_{ijt_{ij}}=\left[z_{ij1},\;z_{ij2},\ldots ,\;z_{ijt_{ij}}\right]$ *denote a vector consisting of power output values, where* $i\in {ℛ}_{>0}$ *is a specific wind speed value*, j=1,…,11 *represents the* *j*-th *WT*, $t_{ij}\in {\mathbb{N}}_{>0}$ (${\mathbb{N}}_{>0}$: *set of positive integers) is the total amount of power output* *values of the* *j*-th *WT* *at* i m/s, and $z_{ija}\neq z_{ijb}$ ∀ a≠b, where $a,b\;\in \;{\mathbb{N}}_{>0}^{t_{ij}}$ (${\mathbb{N}}_{>0}^{t_{ij}}=\left\{1,\ldots ,t_{ij}\right\}$). *Thus, the probability is zero that any two or more of the elements of* $Z_{ijt_{ij}}$ *have equal magnitudes. Suppose that* $z_{ij(1)}$ *denotes the element of* $Z_{ijt_{ij}}$ *whose Euclidean distance to the other elements is the shortest*; $z_{ij(2)}$ *denotes the* *element whose Euclidean distance to the other elements is the second shortest; ... and* $z_{ij(t)}$ *denotes the* *element whose Euclidean distance to the other elements is the longest. Then* $z_{ij(1)}<z_{ij\left(2\right)}<\cdots <z_{ij(t)}$ *denotes the original vector* $Z_{ijt_{ij}}$ *after arrangement in increasing order of magnitude, and therefore the three most* *significant points (i.e., the three most significant power output values) at* i m/s are: $z_{ij(1)}$, $z_{ij(2)}$, *and* $z_{ij(3)}$.

**Definition 3**.  *(Three most significant points of the power output of a WT at a specific wind speed value, for the case in which at least two power output values are the same). If in Definition 2* $z_{ija_{u}}=z_{ija_{v}}$*, with* $u,v\;\in \left\{1,\ldots ,t_{ij}\right\}$*, for at least one* $a_{u}\;\neq a_{v}$*, then for example for the case of* l *equal observations* $z_{ij(a_{1})}=\;\cdots =\;z_{ij(a_{l})}$ *and* q *equal observations* $z_{ij(b_{1})}=\;\cdots =\;z_{ij(b_{q})}$ *but* $z_{ij(a_{1})}\;\neq \;z_{ij(b_{1})}$*, the original vector* $Z_{ijt_{ij}}$ *after arrangement in increasing order of magnitude will be denoted by* $z_{ij(1)}<\;\cdots <\;z_{ij\left(a_{1}\right)}=\cdots =\;z_{ij\left(a_{l}\right)}<\cdots <\;z_{ij\left(b_{1}\right)}=\;\cdots =\;z_{ij\left(b_{q}\right)}<\cdots <z_{ij\left(t\right)}$ *for* $a_{l}<b_{1}$*, with* $a_{1}\in \left\{1,\ldots ,t_{ij}-1\right\},\;a_{2}\in \left\{a_{1}+1,\ldots ,t_{ij}-1\right\},\;\ldots ,\;a_{l}\in \left\{a_{l-1}+1,\ldots ,t_{ij}-1\right\},\;b_{1}\in \left\{a_{l}+1,\ldots ,t_{ij}\right\},\;b_{2}\in \left\{b_{1}+1,\ldots ,t_{ij}\right\},\;\ldots ,\;$ *and* $b_{q}\in \left\{b_{q-1}+1,\ldots ,t_{ij}\right\}$*. Therefore, the three most significant power output points at* i m/s *are the elements of one and only one of the following sets:* $\left\{z_{ij(1)},z_{ij\left(2\right)},z_{ij(3)}\right\}$*,* $\left\{z_{ij(1)},z_{ij\left(a_{1}\right)},z_{ij(a_{1})}\right\}$*,* $\left\{z_{ij(a_{1})},z_{ij\left(a_{1}\right)},z_{ij(a_{1})}\right\}$*,* $\left\{z_{ij(a_{1})},z_{ij\left(a_{1}\right)},z_{ij(3)}\right\}$*,* $\left\{z_{ij(a_{1})},z_{ij\left(a_{1}\right)},z_{ij(b_{1})}\right\}$*,* $\left\{z_{ij(a_{1})},z_{ij\left(b_{1}\right)},z_{ij(b_{1})}\right\}$.

Third, in order to compare the power output of each WT with its GPC, it was necessary to choose the important information that was needed to build the wind speed interval of interest (*I*), in agreement with the wind speed interval of the GPC for the WTs of the VWF. The wind speed interval of this GPC was [3 m/s, 25 m/s], where the measurement points were {3 m/s, 3.5 m/s, 4 m/s, 4.5 m/s, …, 25 m/s} (*i.e.*, 45 points from 3 m/s up to 25 m/s, the step size was equal to 0.5 m/s). However, it was found that after the second step there were not any significant power output points for the wind speed interval from 22 m/s onwards, because in that region most of the power output values were the result of anomalous information. Therefore, the power output information that fell in this latter interval was discarded. In addition, as the electricity production of the WTs at very low wind speeds is meaningless, the power output information in the interval [0 m/s,2.4 m/s) was discarded as well. Thus, only the information on the most significant points that were found in the second step and fell in the interval $I_{0}=\left[2.4\;m/s,22\;m/s\right]$ was used to build the wind speed interval of interest *I*.

Fourth, the most significant points of $I_{0}$ were found in accordance with Definition 4. However, in order to understand Definition 4, it is important to make the following assumptions:

1. Let $M\subset {ℛ}^{2}$ (${ℛ}^{2}$: two-dimensional vector space over the set or real numbers) be the set of the most significant points that were found in the second step and fell in $I_{0}$.
2. Let $m_{i}=\left(m_{i1},m_{i2}\right)\in M,\;i\in {\mathbb{N}}_{>0}$, be the *i*-th point of M, where $m_{i1}$ and $m_{i2}$ are the wind speed and power output value, respectively.
3. Let $w=\left[w_{0},w_{0}+0.2\;m/s\right]$ be a wind speed interval of length 0.2 m/s.
4. Let $M_{j}\subset M,\;j\in {\mathbb{N}}_{>0}^{\leq k}$, be the j-th subset of points of M that fall in the j-th wind speed subinterval of $I_{0}$ (*i.e.*, $I_{0j}\subset I_{0}$) that is created as result of the intersection of $w=\left[w_{0},w_{0}+0.2\;m/s\right]$ with $I_{0}=\left[2.4\;m/s,22\;m/s\right]$ as we increase $w_{0}$ from 2.4 m/s up to 21.8 m/s with a step size of 0.2 m/s. Note that the overlap between adjoint subintervals is equal to 0.1 m/s.
5. Let $m_{j}\in M_{j}$ be the point whose Euclidean distance to the other points of $M_{j}$ is the shortest.

**Definition 4**.  *(Most significant points of* $I_{0}$). *Taking into consideration the above assumptions, the most significant points of* $I_{0}$ *are the points* $m_{j},\;j=1,\;\ldots ,\;k$.

**Figure 4:**
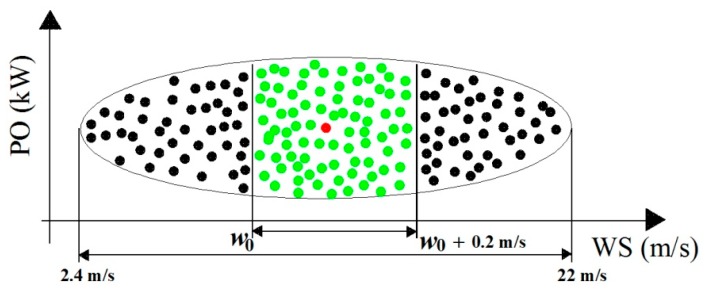
$I_{0j}$: Intersection of $w=\left[w_{0},w_{0}+0.2\;m/s\right]$ with $I_{0}=\left[2.4\;m/s,22\;m/s\right]$. Most significant point of $I_{0j}$: Red o; Points of $I_{0j}$: {Red o, Green o}; Points of the interval $I_{0}$: {Black o, Green o, Red o}. PO: Power Output. WS: Wind Speed.

In order to contribute to a better understanding of the process described in the fourth step, Figure 4 shows a graphical example of the process of formation of interval $I_{0j}$ and its most significant point, $m_{j}$.

Fifth, once the most significant points of $I_{0}$ were found, the interval of interest *I* was built selecting the most significant points of $I_{0}$ that fell in the interval I=[3 m/s,21.5 m/s]. The choice of the lower endpoint of *I* is self-evident and the choice of the upper endpoint of *I* was made in order to be conservative.

Sixth, and finally, the points of the interval *I* were used to build the power curve of the WT for which they were found.

**Figure 5:**
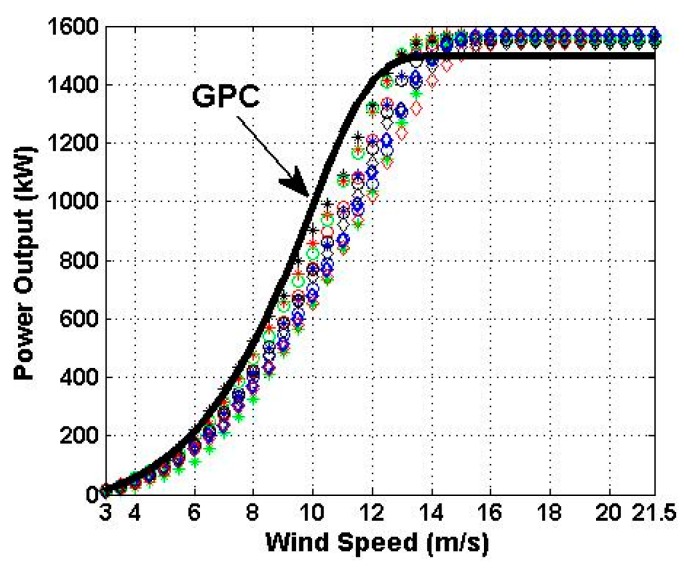
Power curves of the wind turbines (WTs) of the Villonaco Wind Farm in the year 2014, and the Guaranteed Power Curve (GPC). WT-1: Blue *; WT-2: Red *; WT-3: Black *; WT-4: Green *; WT-5: Blue o; WT-6: Red o; WT-7: Black o; WT-8: Green o; WT-9: Blue ⋄; WT-10: Red ⋄; WT-11: Black ⋄.

Figure 5 shows the power curves of the 11 WTs that were compared with the GPC at the measurement points in the interval [3 m/s,21.5 m/s]. The power performance verification is carried out in Section 6.2.

### 6.2. Power Performance Verification

The procedure to carry out the wind farm power performance verification was as explained in Section 4. Table 1 shows the Guaranteed Power Curve. In accordance with what has been stated in Section 6.1, the measurement points are the wind speed values shown in the first and third columns of Table 1.

Here, the statistic *S* (see Equation (2)) was S=194.37 and *p*-value = $P(S\geq 194.37|H_{0}\;\mathrm{is}\;\mathrm{true})=1.0929\times 10^{-35}$, which is the probability of obtaining the observed sample results, or more extreme results, when the null hypothesis $H_{0}$ (see Equation (1)) is actually true. Also, for α=0.05 the critical value [24] was $s_{0.05}=19.6751$ and for α=0.01 the critical value was $s_{0.01}=24.7250$. Therefore, since $S>s_{0.01}>s_{0.05}$, $H_{0}$ was rejected at the α=0.05 significance level and at the α=0.01 significance level as well. In other words, since *p*-value = $1.0929\times 10^{-35}<0.01<0.05,$ $H_{0}$ cannot be accepted. For this reason, in accordance with Theorem 1, it cannot be said that the power performance of the VWF was perfect.

At this point, it is important to say that usually researchers use a significance level of either 0.05 or 0.01 [37,38]. Nevertheless, other significance levels can also be used. However, despite the fact that in this paper it was decided to show the results at both levels (*i.e.*, at the 0.05 level and at the 0.01 level), as it will be seen shortly, the final decision about the power performance of the VWF was made at the 0.01 level, because at this significance level the results are highly significant.

Now, due to the fact that the null hypothesis was rejected, in accordance with what was explained in Section 4, it is important to compare the power performance of the wind turbines to determine which wind turbines produced either a superior outcome or an inferior outcome. In order to do this, the procedure explained in Section 5.1 was used.

Here, taking into consideration the information given in Table 1, we ranked the 11 observations of the wind turbines along with the one of the guaranteed power curve from 1 to 12, for each one of the 38 measurement points (see Table 1). As was already mentioned in Section 5.1, these ranks were called $r_{ij}$, $i=1,\;\ldots ,\;n_{j}$ and j=1, …, k, where for the problem at hand k=12 and $n_{j}=38$ for all j∈{1,…,12}.

Next, the parameters given by Equations (4)–(9) were calculated and the set of all pairwise comparisons given by Equation (10) was built. Afterwards, following the steps described in Section 5.1, at the α=0.05 significance level, it was found that the pairs that did not have a significantly different power performance were the ones shown in Table 2, where WT*i* is the *i*-th wind turbine for i∈{1, …, 11} and WT12 is the guaranteed power curve. In addition, at the α=0.01 significance level, which means that the results are highly significant, it was found that the pairs that did not have a significantly different power performance were the ones shown in Table 3.

**Figure 6:**
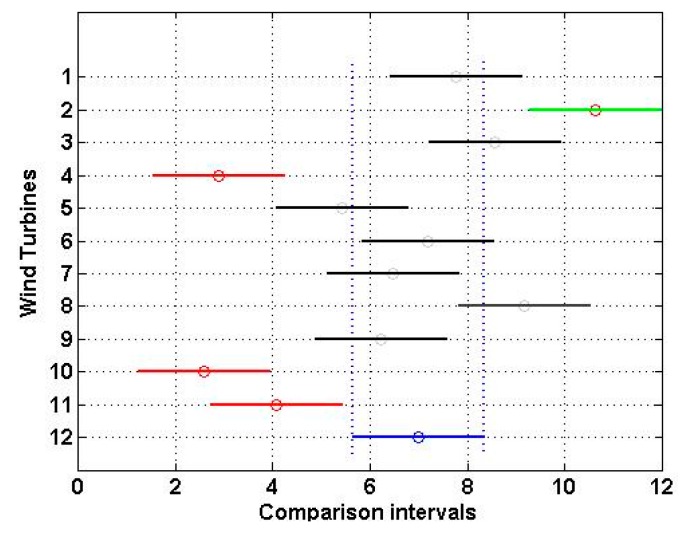
Multiple comparison of the power performance of the wind turbines at the α=0.05 significance level. WT1: Wind Turbine 1 (Black); WT2: Wind Turbine 2 (Green); WT3: Wind Turbine 3 (Black); WT4: Wind Turbine 4 (Red); WT5: Wind Turbine 5 (Black); WT6: Wind Turbine 6 (Black); WT7: Wind Turbine 7 (Black); WT8: Wind Turbine 8 (Black); WT9: Wind Turbine 9 (Black); WT10: Wind Turbine 10 (Red); WT11: Wind Turbine 11 (Red); WT12: Guaranteed Power Curve (Blue).

These results show that the power performance of the VWF was not perfect (see Theorem 1). Figure 6 shows the multiple comparison (see Equation (11)) of the power performance of the wind turbines at the α=0.05 significance level. That is to say, this figure shows the multiple comparison of the means $\bar{R_{\cdot 1}},\;\ldots ,\;\bar{R_{\cdot 12}}\;$given by Equation (5), at the α=0.05 significance level, where these means are the center points of each of the 12 horizontal segments, and each comparison interval is represented by the projection of the endpoints of each one of these 12 segments on the horizontal axis of the figure.

In Figure 6, the comparison interval of the guaranteed power curve (WT12) is in blue (dashed lines). If the comparison interval of any wind turbine does not intersect with the interval for WT12, it can be said that their power performances are significantly different from each other. From Figure 6, it can be seen that the power performance of wind turbines 2, 4, 10, and 11 (*i.e.*, WT2, WT4, WT10, and WT11, respectively) was significantly different from the power performance of WT12 (*i.e.*, the guaranteed power curve) at the α=0.05 significance level. However, it is important to point out that the power performance of WT2 was significantly better than the one of WT12.

**Figure 7:**
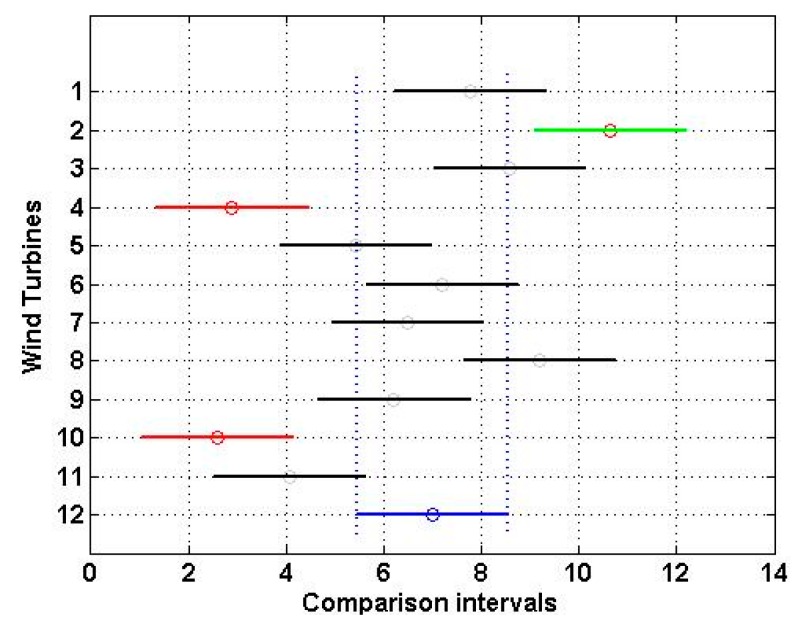
Multiple comparison of the power performance of the wind turbines at the α=0.01 significance level. WT1: Wind Turbine 1 (Black); WT2: Wind Turbine 2 (Green); WT3: Wind Turbine 3 (Black); WT4: Wind Turbine 4 (Red); WT5: Wind Turbine 5 (Black); WT6: Wind Turbine 6 (Black); WT7: Wind Turbine 7 (Black); WT8: Wind Turbine 8 (Black); WT9: Wind Turbine 9 (Black); WT10: Wind Turbine 10 (Red); WT11: Wind Turbine 11 (Black); WT12: Guaranteed Power Curve (Blue).

Furthermore, Figure 7 shows the multiple comparison of the power performance of the wind turbines at the α=0.01 significance level. As in Figure 6, in Figure 7 the comparison interval of the guaranteed power curve (WT12) is in blue (dashed lines). If the comparison interval of any wind turbine does not intersect with the interval for WT12, it can be said that their power performances are significantly different from each other.

From Figure 7, it can be seen that the power performance of WT2, WT4, and WT10 was significantly different from the power performance of WT12 at the α=0.01 significance level. Also, once again, the power performance of WT2 was significantly better than that of WT12.

Finally, taking into consideration Definition 1 and the results shown in Figure 5 and Figure 7, it can be said that the power performance of the Villonaco Wind Farm is acceptable.

## 7. Conclusions

In this paper, a nonparametric method of verification of the power performance of a wind farm has been presented. The proposed method is based on the Friedman’s test; it only requires the data collected by the SCADA system, and is independent of the distribution of the population from which the samples are drawn.

Here, the guaranteed power curve of the wind turbines has been used as one more wind turbine of the wind farm under assessment. The results of the nonparametric statistical analysis of the experimental data, which were collected by the SCADA system for a year of operation of the wind farm, showed that the power performance of the wind farm was acceptable. In short, the wind farm under assessment consists of 11 wind turbines and it has been shown that the power performance of eight of these wind turbines is as expected, and the power performance of one of them is much better than expected. This was wind turbine 2. Also, from Figure 5 and Figure 7 it can be seen that the power performance of wind turbines 4 and 10 can be easily improved by carrying out some minor technical adjustments, in order to improve their power curve in the approximate interval of wind speed from 10 m/s up to 14 m/s. The method presented in this paper is inexpensive, easy to apply, and contributes to enrich actual techniques that exist to verify the power performance of wind farms.

## Figures and Tables

**Table 1:** Guaranteed power curve for the wind speed interval [3 m/s,21.5 m/s]. WS: Wind Speed. PO: Power Output.

WS at Hub Height (m/s)	PO (kW)	WS at Hub Height (m/s)	PO (kW)
3	16	12.5	1455
3.5	31	13	1481
4	55	13.5	1494
4.5	85	14	1500
5	121	14.5	1500
5.5	161	15	1500
6	211	15.5	1500
6.5	273	16	1500
7	345	16.5	1500
7.5	425	17	1500
8	514	17.5	1500
8.5	616	18	1500
9	729	18.5	1500
9.5	854	19	1500
10	984	19.5	1500
10.5	1114	20	1500
11	1234	20.5	1500
11.5	1334	21	1500
12	1409	21.5	1500

**Table 2:** Pairs of wind turbines (WTs) that did not have a significantly different power performance at the α=0.05 significance level.

	**WT1**	**WT2**	**WT3**	**WT4**	**WT5**	**WT6**	**WT7**	**WT8**	**WT9**	**WT10**	**WT11**	**WT12**
**WT1**	X		X		X	X	X	X	X			X
**WT2**		X	X					X				
**WT3**	X	X	X			X	X	X	X			X
**WT4**				X	X					X	X	
**WT5**	X			X	X	X	X		X		X	X
**WT6**	X		X		X	X	X	X	X			X
**WT7**	X		X		X	X	X		X		X	X
**WT8**	X	X	X			X		X				X
**WT9**	X		X		X	X	X		X		X	X
**WT10**				X						X	X	
**WT11**				X	X		X		X	X	X	
**WT12**	X		X		X	X	X	X	X			X

**Table 3:** Pairs of wind turbines (WTs) that did not have a significantly different power performance at the α=0.01 significance level.

	**WT1**	**WT2**	**WT3**	**WT4**	**WT5**	**WT6**	**WT7**	**WT8**	**WT9**	**WT10**	**WT11**	**WT12**
**WT1**	X	X	X		X	X	X	X	X			X
**WT2**	X	X	X					X				
**WT3**	X	X	X			X	X	X	X			X
**WT4**				X	X					X	X	
**WT5**	X			X	X	X	X		X	X	X	X
**WT6**	X		X		X	X	X	X	X			X
**WT7**	X		X		X	X	X	X	X		X	X
**WT8**	X	X	X			X	X	X	X			X
**WT9**	X		X		X	X	X	X	X		X	X
**WT10**				X	X					X	X	
**WT11**				X	X		X		X	X	X	X
**WT12**	X		X		X	X	X	X	X		X	X

## References

[1] IEC 61400 (2005). Part 12–1: Power Performance Measurements of Electricity Producing Wind Turbines.

[2] Oh H., Kim B. (2015). Comparison and verification of the deviation between guaranteed and measured wind turbine power performance in complex terrain. Energy.

[3] Smith B., Link H., Randall G., McCoy T. Applicability of nacelle anemometer measurements for use in turbine power performance tests. Proceedings of the American Wind Energy Association (AWEA) WINDPOWER.

[4] Butler S., Ringwood J., O’Connor F. Exploiting SCADA system data for wind turbine performance monitoring. Proceedings of the Conference on Control and Fault-Tolerant Systems (SysTol).

[5] Kusiak A., Zheng H., Song Z. (2009). Models for monitoring wind farm power. Renew. Energy.

[6] Kusiak A., Zheng H., Song Z. (2009). On-line monitoring of power curves. Renew. Energy.

[7] Kusiak A., Verma A. (2013). Monitoring wind farms with performance curves. IEEE Trans. Sustain. Energy.

[8] Yang W., Court R., Jiang J. (2013). Wind turbine condition monitoring by the approach of SCADA data analysis. Renew. Energy.

[9] Zolfaghari S., Riahy G.H., Abedi M. (2015). A new method to adequate assessment of wind farms’ power output. Energy Convers. Manag..

[10] Shokrzadeh S., Jozani M.J., Bibeau E. (2014). Wind turbine power curve modeling using advanced parametric and nonparametric methods. IEEE Trans. Sustain. Energy.

[11] Lydia M., Selvakumar A.I., Kumar S.S., Kumar G.E.P. (2013). Advanced algorithms for wind turbine power curve modeling. IEEE Trans. Sustain. Energy.

[12] Schlechtingen M., Santos I.F., Achiche S. (2013). Using data-mining approaches for wind turbine power curve monitoring: A comparative study. IEEE Trans. Sustain. Energy.

[13] Chen N., Qian Z., Nabney I.T., Meng X. (2014). Wind power forecasts using Gaussian processes and numerical weather prediction. IEEE Trans. Power Syst..

[14] Santos P., Villa L.F., Reñones A., Bustillo A., Maudes J. (2015). An SVM-based solution for fault detection in wind turbines. Sensors.

[15] Lee G., Ding Y., Genton M.G., Xie L. (2015). Power Curve Estimation With Multivariate Environmental Factors for Inland and Offshore Wind Farms. J. Am. Stat. Assoc..

[16] Jeon J., Taylor J.W. (2012). Using conditional kernel density estimation for wind power density forecasting. J. Am. Stat. Assoc..

[17] Cheng L., Lin J., Sun Y.Z., Singh C., Gao W.Z., Qin X.M. (2012). A model for assessing the power variation of a wind farm considering the outages of wind turbines. IEEE Trans. Sustain. Energy.

[18] Ganger D., Zhang J., Vittal V. (2014). Statistical characterization of wind power ramps via extreme value analysis. IEEE Trans. Power Syst..

[19] Cai D., Shi D., Chen J. (2014). Probabilistic load flow computation using copula and Latin hypercube sampling. IET Gener. Transm. Distrib..

[20] Xie K., Li Y., Li W. (2012). Modelling wind speed dependence in system reliability assessment using copulas. IET Renew. Power Gener..

[21] Wang Y., Infield D.G., Stephen B., Galloway S.J. (2014). Copula-based model for wind turbine power curve outlier rejection. Wind Energy.

[22] Gill S., Stephen B., Galloway S. (2012). Wind turbine condition assessment through power curve copula modeling. IEEE Trans. Sustain. Energy.

[23] Lydia M., Suresh Kumar S., Immanuel Selvakumar A., Edwin Prem Kumar G. (2014). A comprehensive review on wind turbine power curve modeling techniques. Renew. Sustain. Energy Rev..

[24] Hollander M., Wolfe D.A., Chicken E. (2014). Nonparametric Statistical Methods.

[25] Friedman M. (1937). The use of ranks to avoid the assumption normality implicit in the analysis of variance. J. Am. Stat. Assoc..

[26] Hernandez W., Maldonado-Correa J.L., Méndez A. (2016). Frequency-domain analysis of performance of a wind turbine. Electron. Lett..

[27] Robalino-López A., Mena-Nieto A., García-Ramos J.E. (2014). System dynamics modeling for renewable energy and CO_2_ emissions: A case study of Ecuador. Energy Sustain. Dev..

[28] Lee M., Lee S., Hur N., Choi C.-K. A numerical simulation of flow field in a wind farm on complex terrain. Proceedings of the Seventh Asia-Pacific Conference on Wind Engineering (APCWE-VII).

[29] Polanco G., Shakeel Virk M. Role of advanced CAE tools in the optimization of wind resource assessment of complex terrains. Proceedings of the 4th IEEE International Conference on Cognitive Infocommunications (CogInfoCom 2013).

[30] Xu C., Yang J., Li C., Shen W., Zheng Y., Liu D. A research on wind farm micro-sitting optimization in complex terrain. Proceedings of the 2013 International Conference on Aerodynamics of Offshore Wind Energy Systems and Wakes (ICOWES2013).

[31] Marti I., San Isidro M.J., Cabezón D., Loureiro Y., Villanueva J., Cantero E., Pérez I. Wind power prediction in complex terrain: From synoptic scale to the local scale. Proceedings of the Science of Making Torque from Wind.

[32] Gasch R., Twele J. (2012). Wind Power Plants.

[33] Burton T., Jenkings N., Sharpe D., Bossanyi W. (2011). Wind Energy Handbook.

[34] Sainz E., Llombart A., Guerrero J.J. (2009). Robust filtering for the characterization of wind turbines: Improving its operation and maintenance. Energy Convers. Manag..

[35] Hochberg Y., Tamhane A.C. (1987). Multiple Comparison Procedures.

[36] Meteodyn Metrology & Dynamics. http://www.meteodyn.com.

[37] Craparo R.M., Salkind N.J. (2007). Significance level. Encyclopedia of Measurement and Statistics, Volume 3.

[38] Sproull N.L. (2003). Handbook of Research Methods: A Guide for Practitioners and Students in the Social Science.

